# Copper and Temperature Interactions Induce Differential Physiological and Metal Exclusion Responses in the Model Brown Macroalga *Ectocarpus*

**DOI:** 10.3390/plants14121834

**Published:** 2025-06-14

**Authors:** Alex Santillán-Sarmiento, Paula S. M. Celis-Plá, A. John Moody, Claudio A. Saez, Murray T. Brown

**Affiliations:** 1 Research Center for Territory and Sustainable Habitat, Facultad de Ciencias del Medio Ambiente, Universidad Tecnológica Indoamérica, Quito 170103, Ecuador; 2 Laboratory of Coastal Environmental Research, HUB AMBIENTAL UPLA, Facultad de Ciencias Naturales y Exactas, Universidad de Playa Ancha, Valparaíso 2360004, Chile; paulacelispla@upla.cl (P.S.M.C.-P.); claudio.saez@ua.es (C.A.S.); 3 School of Biological and Marine Sciences, University of Plymouth, Drake Circus, Plymouth PL4 8AA, UK; 4 Departamento de Ciencias del Mar y Biología Aplicada, Universidad de Alicante, 03690 Alicante, Spain

**Keywords:** climate change, seaweed, trace metals, chlorophyll *a* fluorescence, rapid light curves

## Abstract

The toxic effects of copper (Cu) excess in brown macroalgae have been well characterized. However, the interactive effects of increased temperatures, associated with climate change, and Cu stress on these macrophytes remain almost unexplored. In this study, we exposed the model brown seaweed *Ectocarpus* to different Cu concentrations (0, 0.8, 1.6, and 3.2 μM) at two different temperatures (15 and 25 °C). Relative growth rates decreased at 25 °C for the two highest Cu concentrations after 8 days of exposure, but a contrasting pattern was observed in the photosynthetic maximum quantum yield (*F_v_*/*F_m_*) and photosynthetic efficiency (*α*), where reductions were observed at 15 °C for the same Cu concentrations. Although no differences among treatments were observed for chlorophyll *a* (Chl*a*) and chlorophyll *c* (Chl*c*), a reduction in concentration of the accessory pigment fucoxanthin (Fx) was only observed at 15 °C in all Cu treatments. Interestingly, at 25 °C, 20.1% less total Cu (intracellular + extracellularly bound) accumulated compared to 15 °C upon exposure to 3.2 μM Cu. Likewise, 33.1 and 23.8% less Cu accumulated intracellularly at 25 °C after exposure to 1.6 μM and 3.2 μM Cu, respectively. Additionally, at 25 °C about half of the Cu ions accumulated intracellularly and half extracellularly compared to 15 °C, where Cu accumulated mostly intracellularly at the two highest Cu concentrations. The results presented here provide valuable information to better understand the interactive effects of increased temperature and excess Cu in the stress response of *Ectocarpus*, suggesting that increased temperature helps to offset the negative impacts of exposure to high Cu concentrations.

## 1. Introduction

Marine macroalgae (seaweeds) are important primary producers occurring in coasts and estuaries worldwide providing crucial ecological and economic services to the communities associated with them [1,2,3]. Some seaweed taxa are key habitat structuring agents functioning as ecosystem engineers, which harbor a high diversity of marine organisms [4,5]. Such characteristics link them culturally and economically to humans through the provision of ecosystem goods and services, ranging from food to medicine and storm protection [6]. Numerous species of macroalgae thrive in the intertidal and subtidal zones, where they are exposed to daily and seasonal abiotic pressures such as desiccation, variations in salinity, UV radiation, and temperature fluctuations [7,8]. Those pressures are predicted to be altered by ongoing climate change on a global scale, potentially generating stressful conditions for marine organisms including seaweed [9,10]. Furthermore, the occurrence of other anthropogenic stressors on the local scale, such as pollution by metals and pesticides, could elicit interactions among stressors, with unpredictable consequences for the biota [11,12].

One of the most perceptible effects of climate change is the rapid increase in atmospheric and oceanic temperatures, which has raised concerns about its direct and indirect implications in the long-term for marine life [13]. Some authors suggested that sea surface temperatures (SSTs) could increase on average of 2–4 °C by year 2100 [10]. However, more immediate threats are posed by increasingly common marine heat waves (MHWs), which are abnormally high ocean temperatures that can affect vast ocean areas and last for weeks to years [14]. Those increases are expected to differently affect seaweed species according to their range of tolerances [15,16]. For example, it was observed that species occurring in environments with low seasonal temperature fluctuations, such as the tropics and Antarctica, are less tolerant to temperature alterations compared to species thriving in temperate regions, where seasonal fluctuations are more conspicuous [17,18,19,20]. When the temperature is high enough to surpass seaweed’s tolerance capacity, the disruption of cellular processes may take place. For instance, long-term exposure to disruptive temperatures may impair vital cellular processes such as photosynthesis and enzymatic activity [21,22]. The main temperature-sensitive sites in the photosynthetic apparatus are suggested to be photosystem II (PSII) as well as the oxygen-evolving complex, ATP synthase, and enzymes in the Calvin cycle [23]. In early studies, Davison [24] found that photosynthetic rates were inversely proportional to growth temperature as well as the activity of ribulose-l,5-bisphosphate carboxylase/oxygenase (RuBisCO) in *Saccharina latissima*. Additionally, photosystem II (PSII) is directly affected by temperature-induced changes in the thylakoid membrane fluidity, displacing PSII light-harvesting complexes from the thylakoid membrane [23]. Indirectly, membranes and enzymes can be affected by reactive oxygen species (ROS) produced under stressful temperatures [25]. Although the temperature tolerance range of some seaweeds has been assessed [22], interactions with other stressors at the local scale are usually not considered. However, it is necessary to understand these interactions to assist with the efforts on ecosystem management [26].

On a local scale, seaweeds are exposed to specific pressures such as pollution by metals. For instance, copper (Cu) is a well-characterized source of stress for macroalgae in sites affected by human activities [27]. Despite its essential role as a micronutrient at low concentrations, Cu is highly toxic and an effective inhibitor of growth in brown, red, and green seaweeds when concentrations exceed certain thresholds [28,29,30]. The mechanisms of Cu toxicity involve an impairment of photosynthetic physiology [31,32,33]. For example, Cu was able to substitute the central atom of magnesium in the chlorophyll molecules of brown seaweed *Ectocarpus* sp. [34], forming non-functional chlorophyll molecules that were not capable of light harvesting [33,34,35]. This was also suggested to be the reason for decreases in basal (*F_o_*) and maximal (*F_m_*) chlorophyll *a* fluorescence in non-tolerant populations of *Fucus serratus* exposed to Cu up to 2 µM [36]. Seaweeds readily accumulate Cu in their cell walls and intracellularly, but this distinction has rarely been studied [37]. There is evidence that, in non-polluted sites, most of the Cu accumulates intracellularly [38], whereas in seaweeds growing in Cu-impacted sites, more is accumulated extracellularly [39]. This exclusion mechanism may account for the resistance capacity of some species in heavily polluted environments. Additionally, there is a strong relationship between changes in temperature and the bioavailability of Cu species to algae [40,41]. Thus, increased temperature due to climate change might modulate the metal uptake capacity of seaweeds by either altering the biochemical processes behind exclusion mechanisms or by reducing availability due to altered speciation. Therefore, understanding the potential interactions between a global stressor (e.g., ocean warming) and a local stressor (e.g., Cu pollution) and their effects on the (eco-)physiology of seaweeds has become highly important.

Although there are studies addressing the interactions between temperature and other stressors such as excess UV radiation in seaweeds [42,43,44], studies assessing interactions of metals and other abiotic stressors are rare. In the present study, we examined the interactions between increased temperature and Cu excess on the response of the brown algae *Ectocarpus* sp. (formerly *Ectocarpus siliculosus* (Dillwyn) Lyngbye, henceforth referred as *Ectocarpus*) in laboratory conditions. This filamentous seaweed has become a model for the study of brown algae (Phaeophyceae) due to its ease of cultivation in the laboratory, availability of its genome, and its close evolutionary relationship with the economically and ecologically important orders Laminariales and Fucales [45]. These characteristics make *Ectocarpus* an organism of choice for assessing in-depth aspects of seaweed stress physiology. We hypothesized differences in the interactions between stress factors (different levels of copper and increased temperature conditions) in the response of *Ectocarpus*, evaluated by measuring the relative growth rate, photosynthetic performance, pigment composition, and Cu accumulation capacity.

## 2. Results

### 2.1. Growth

**Figure 1:**
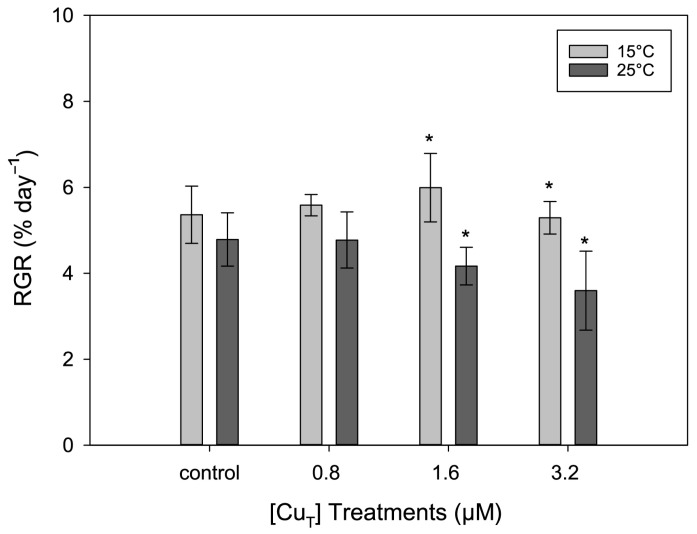
Relative growth rates (RGRs) of *Ectocarpus* exposed to combinations of one of four Cu concentrations (0, 0.8, 1.6, and 3.2 μM) and one of two temperatures (15 °C and 25 °C) for 8 days. Asterisks (*) indicate significant differences (*p* < 0.05) found after Tukey’s test for factor ‘temperature’. Error bars represent ± 1 SD, *n* = 3.

At both temperatures there was no significant change in the RGR in response to Cu exposure (*p* = 0.199). This was reflected in a non-significant interaction term ‘Cu × temperature’ (*p* = 0.252) (Table S1a). However, at the two highest Cu concentrations, there were significant (*p* < 0.05) decreases in the RGR at 25 °C (Figure 1). In contrast, under the control (no added Cu) and the lowest Cu concentration (0.8 μM), reductions in RGR at 25 °C were not significantly different.

### 2.2. Photosynthetic Performance 

**Figure 2:**
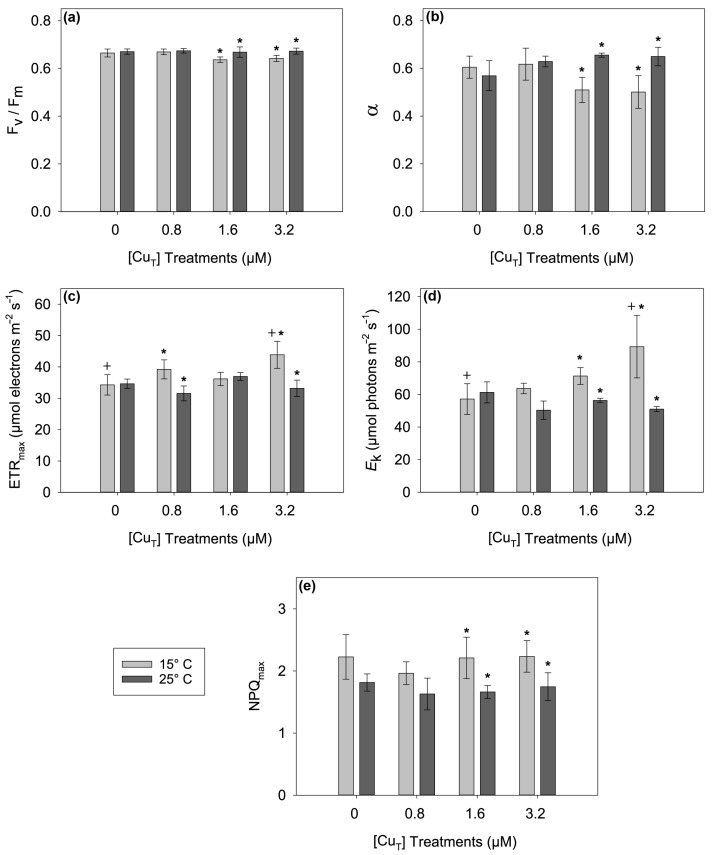
Photosynthetic parameters: (**a**) *F_v_*/*F_m_*: maximum quantum yield, (**b**) *α*: efficiency of light captured for photosynthesis, (**c**) *ETR_max_*: maximum electron transport rate, (**d**) *E*_k_: minimum saturating irradiance, (**e**) *NPQ_max_*: maximal non-photochemical quenching, obtained from rapid light curves (RLCs) in *Ectocarpus* exposed to combinations of four Cu concentrations (0, 0.8, 1.6, and 3.2 μM) and two temperatures (15 °C and 25 °C) for 6 days. Asterisks (*) indicate significant differences (*p* < 0.05) found after Tukey’s test for factor ‘temperature’ within Cu concentration, and crosses (+) for factor ‘copper’ within temperature. Error bars indicate ± 1 SD, *n* = 3.

The interactions between Cu and temperature were observed in all the photosynthetic parameters derived from the rapid light curves of *ETR* versus irradiance (i.e., *α*, *ETR_max_*, and *E*_k_; *p* < 0.05) but not for the maximum quantum yield (*F_v_*/*F_m_*) or maximum non-photochemical quenching (*NPQ_max_*) (Table S1b). *F_v_*/*F_m_* was lower at 15 °C compared to 25 °C after treatment with 1.6 and 3.2 μM Cu but not after treatment with the lowest Cu concentration (0.8 μM) or in the control (Figure 2a). The parameter *α* is an indicator of the light capture efficiency and showed a response similar to *F_v_*/*F_m_*; however, in this case an interaction between Cu and temperature was observed (*p* < 0.05) (Figure 2b). For the maximum electron transport rate or *ETR_max_* and the minimum saturating irradiance or *E*_k_, the response to Cu was dependent on the growth temperature being greater at 15 °C after treatment with 3.2 μM compared to 25 °C after the same Cu treatment (Figure 2c,d). For *NPQ_max_*, the only significant differences were between 25 °C and 15 °C after treatment with 1.6 and 3.2 μM Cu (*p* < 0.05) (Figure 2e).

### 2.3. Photosynthetic Pigments Content

**Figure 3:**
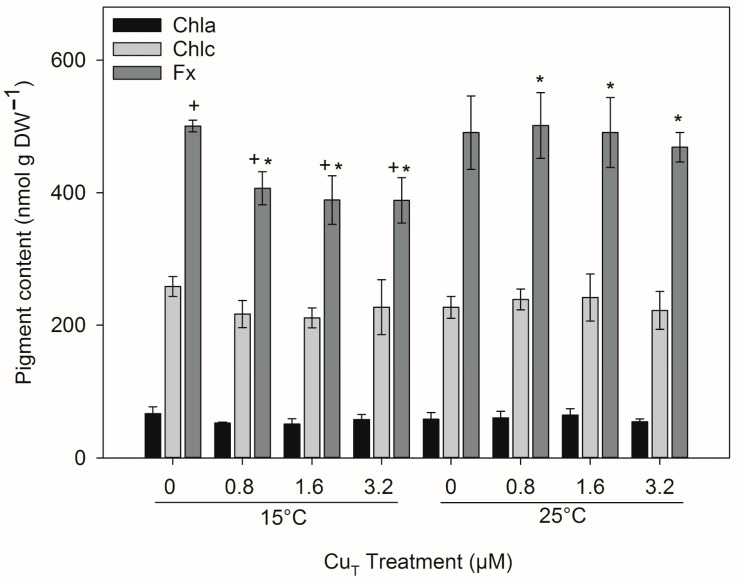
Concentrations of Chl*a*, Chl*c*, and Fx in *Ectocarpus* exposed to a combination of one of four Cu concentrations (0, 0.8, 1.6, and 3.2 μM) and one of two temperatures (15 °C and 25 °C) for 6 days. Asterisks (*) indicate significant reductions in Fx content (*p* < 0.05) found after Tukey’s test for factor ‘temperature’ and crosses (+) for factor ‘copper’. Error bars indicate ± 1 SD, *n* = 3.

There were no statistical differences in the concentrations of Chl*a* and Chl*c* (Figure 3) between any treatments following 6 days of exposure (Table S1c). In contrast, the concentrations of the accessory pigment Fx were significantly reduced at 15 °C after treatment with 0.8, 1.6, and 3.2 μM (*p* < 0.05), while no difference was observed at 25 °C between Cu treatments. No statistically significant interaction was observed between the factors for Fx.

### 2.4. Intra- and Extracellular Cu Accumulation

**Figure 4:**
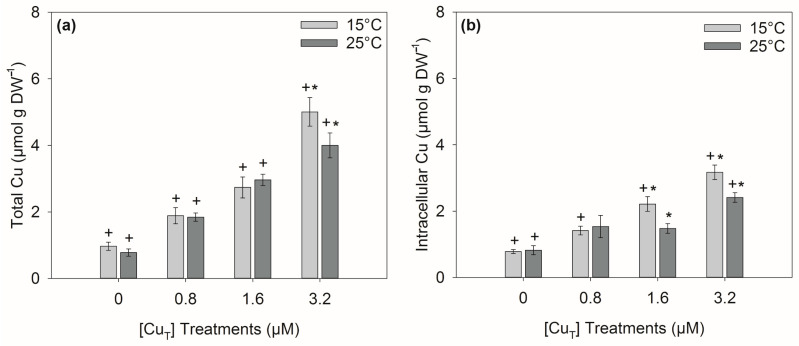
Total (**a**) and intracellular (**b**) copper accumulation in *Ectocarpus* exposed to combinations of one of four Cu concentrations (0, 0.8, 1.6, and 3.2 μM) and one of two temperatures, 15 °C and 25 °C, for 6 days. Asterisks (*) indicate significant differences (*p* < 0.05) found after Tukey’s test for factor ‘temperature’ within Cu concentration and crosses (+) for factor ‘copper’ within temperature. Error bars indicate ± 1 SD, *n* = 3.

A significant interaction for Cu treatment × temperature was observed for both ‘total Cu’ accumulation (intracellular + extracellularly bound; *p* < 0.05) and for ‘intracellular Cu’ accumulation (Table S1d). After exposure to 3.2 μM, 20.1 % less ‘total Cu’ accumulated at 25 °C (*p* < 0.05) compared to total accumulation at 15 °C, but, after exposure to low and medium Cu (0.8 and 1.6 μM), the levels of accumulation were similar at both temperatures (Figure 4a). Likewise, 33.1 and 23.8% less Cu accumulated intracellularly (*p* < 0.05) at 25 °C after exposure to 1.6 μM and 3.2 μM, respectively, but similar levels were observed at low Cu exposure (0.8 μM) and in the control (Figure 4b). At 25 °C, about half of the Cu ions were bound extracellularly and half intracellularly after treatment with 1.6 μM and 3.2 μM Cu (Table 1). In contrast, at 15 °C proportionally more Cu accumulated intracellularly after treatment with 1.6 and 3.2 μM Cu (Table 1).

## 3. Discussion

In this study, we found that interactions between excess Cu and increased temperature produced different responses in each endpoint measured: growth, photosynthetic performance, and Cu accumulation in *Ectocarpus*. The interactions between the stress factors were absent for relative growth rate (RGR), *F_v_*/*F_m_*, *NPQ_max_*, and pigment content, while interactions between Cu and temperature were observed for *α*, *ETR_max_*, *E*_k_, and for copper accumulation (Table S1).

Water temperature plays a key role in the growth and physiology of marine macroalgae [22]. The results for the relative growth rate (RGR) indicate that temperature is an important factor for algal growth, as reductions in growth rate ranging from 0.8 to 1.8% per day were observed at the higher temperature of 25 °C regardless of the Cu concentration (Figure 1). In contrast, Major and Davison [46] observed that the embryos of the brown seaweed *Fucus evanescens* were larger when growing at 20 °C compared to 5 °C. Those interspecific differences in the response to temperature may be correlated with the optimal temperature range for growth of the specific species. This seems to be the case in different isolates of *Ectocarpus*, where different optimal growth temperatures have been related to the original collection site [47]. Also, different susceptibilities to temperature have been observed in other Phaeophycean species such as *Ecklonia radiata*, *Scytothalia dorycarpa*, and *Sargassum fallax,* corresponding to latitudinal and temperature gradients [48]. In terms of Cu exposure, there was no significant change in the RGR over the range of Cu concentrations tested in this study. These results agree with those obtained by Sáez et al. [27] and indicate that this strain of *Ectocarpus*, which came from a Cu-polluted site in Chile, is able to resist high Cu concentrations.

The opposite to the RGR was observed for photosynthetic maximum quantum yield: reductions in the *F_v_*/*F_m_* occurred at 15 °C rather than 25 °C upon exposure to the two highest Cu treatments of 1.6 and 3.2 μM (Figure 2a). Thus, a 10 °C increase in temperature compared to the long-term storage temperature (15 °C) did not negatively affect the photosynthetic efficiency, even at high copper concentrations. This phenomenon has also been reported in a similar study for both the germling and adult stages of the brown alga *Fucus serratus* [49]. In that study, *F_v_*/*F_m_* was unaffected in adults and increased in germlings exposed to 1 μM Cu at 22 °C compared to individuals grown at 12 °C. Also, in other photosynthetic organisms such as the green microalga *Chlorella pyrenoidosa*, *F_v_*/*F_m_* was notably reduced at 15 °C even at very low copper concentrations (<0.4 μM) compared to algae incubated at 20, 25, 30, and 35 °C [50]. The light capture efficiency (*α*) was also lower at 15 °C compared to 25 °C after exposure to the two highest Cu concentrations (1.6 and 3.2 μM). This strongly indicates that light harvesting is at least partially responsible for the decrease in *F_v_*/*F_m_*, and it is dependent on the level of Cu exposure as there was a significant interaction between temperature and Cu for *α* (Table S1b). Machalek et al. [51] noted that in *Laminaria saccharina* (*Saccharina latissima*) *α* was affected by temperature, which was dependent on the light adaptation status of the samples prior to taking measurements. The Cu-induced reductions in *α* at 15 °C and high Cu concentrations may indicate an uncoupling in the energy transfer between the antenna and the reaction centers, probably due to an impairment of the light-harvesting capacities of PSII caused by the substitution of Cu^2+^ for Mg^2+^ in the chlorophyll molecules, as suggested by Küpper et al. [33,34,35]. However, the absence of damage under Cu exposure at 25 °C indicates that acclimation or repair mechanisms are activated to deal with the exposure to multiple stressors [49]. In the present study, interactions between both abiotic factors also occurred for the minimum saturating irradiance (*E*_k_) and maximum electron transport rate (*ETR_max_*). These parameters were higher at 15 °C, but the extent of the increase was dependent on the level of Cu exposure. For example, samples exposed to the highest Cu concentration (3.2 μM) showed higher levels of *ETR_max_* at 15 compared to at 25 °C, which helped explain the maintenance of the RGR at 15 °C. However, the increase in *E*_k_ may imply that higher irradiances are necessary to maintain primary metabolism under Cu stress. This is supported by studies on enzymatic activity related to primary metabolism such as RuBisCO in *S. latissima* [24,52], where this enzyme was found to have higher activities at lower temperatures. The high activity of this enzyme was related to the tolerance of this alga to low temperatures, allowing it to maintain growth and high photosynthetic rates. Thus, an elevated *E*_k_ may be indicative of increased primary metabolic reactions in response to stress. Alternatively, the increases in *E*_k_ and *ETR_max_* may imply a temperature-mediated regulation of photosynthesis, allowing faster electron flow along the electron transport chain, thus avoiding possible photoinhibition [53]. Similar results were observed in *Cystoseira tamariscifolia* showing higher *ETR_max_* values during summertime when water temperatures were higher in rocky shores and rockpools [54]. The *NPQ_max_* (heat dissipation indicator) was lower at 25 compared to at 15 °C at 1.6 and 3.2 μM Cu. These results match other results in *Fucus vesciculosus*, where reductions in *NPQ_max_* were also observed at increased temperatures [55]. The higher levels of *NPQ_max_* at 15 °C indicate enhanced heat dissipation as more light is being absorbed (as deduced from the increased *E*_k_) and/or not being used effectively. As a consequence, the energy must be dissipated as heat via mechanisms such as the xanthophyll cycle [56].

The concentrations of chlorophyll (Chl*a* and *c*) were unaffected by either Cu or temperature after 6 days of exposure (Figure 3). Similarly, the concentrations of fucoxanthin (Fx) were not affected at 25 °C under all Cu treatments but were lower at 15 °C. This may be related to alterations in light harvesting as discussed above. Temperature can affect pigment concentrations; for example, *S. latissima* grown at 20 °C compared to 0–5 °C had higher concentrations of Fx, which was associated with increased light-harvesting efficiency [24]. Also, in embryos of *Fucus evanescens*, the Fx content was significantly higher at 20 °C compared with 5 °C due to an increase in PSII reaction center densities [46]. A study on the effects of a combination of increased Cu and temperature in the red alga *Gelidium floridanum* found that exposure to high Cu (3 μM) at 24 °C decreased the levels of Chl*a* [26]. In contrast, at 30 °C the differences between controls and treated samples disappeared, but an overall decrease in pigment concentration was observed after 7 days of exposure. However, after 14 days, concentrations of Chl*a* increased compared to 7 days in all the treatments, and the differences between temperatures under high Cu concentration almost vanished. These findings exemplify how dynamic the response to a combination of stressors can be. Our results for Fx and light-harvesting efficiency (*α*), both of which remained mostly unaltered at 25 °C but lowered at 15 °C after 6 days of exposure, indicate that temperature regulates the tolerance of the photosynthetic apparatus to Cu stress.

In this study, *Ectocarpus* accumulated Cu at both the temperatures assessed. However, increasing the temperature to 25 °C affected the total accumulation of Cu and particularly the intracellular fraction, which was lower at 25 °C than at 15 °C under the 1.6 and 3.2 μM Cu treatments (Figure 4b). This implies that transport and/or Cu exclusion mechanisms were acting at elevated temperatures, as roughly 50% of the total Cu was accumulated extracellularly at 25 °C compared with ca. 80% at 15 °C in the 1.6 μM Cu treatment (Table 1). Some studies suggest that the resistance to excess copper in brown algae may be due to a combination of exclusion and intracellular mechanisms of detoxification (tolerance) [39,57,58]. The strain used in this study was collected from a Cu-polluted site and was considered Cu-resistant due to efficient detoxification mechanisms [27,39,59], so we could hypothesize that the rate of detoxification would be improved at higher temperatures in this strain. However, temperature itself can alter the stability and structure of cell membranes and thus contribute to differences in the uptake of Cu ions due to the alterations in high- and low-affinity Cu transporters such as members of the Ctr family [60,61,62]. Another possibility is that an increase in temperature promoted an enhanced synthesis of organic ligands, such as polyphenols, to detoxify intracellular excess of Cu by chelating and transporting the ligand–metal complexes outside the cell [57,63]. Some data supporting this hypothesis have been provided by Kreusch et al. [26], who found that a reduction in the total phenolic content was driven by the exposure to a high Cu concentration. However, no further reduction was observed between 24 and 30 °C. Additionally, both Cu and temperature have been reported to cause modifications in the cell walls of both algae and plants [64,65]. For instance, a cytophysiological study on *Ectocarpus* strain Es524 demonstrated that 2.4 μM Cu reduced the cell length and led to plasmolysis, and these changes were partially attributed to alterations in the cell wall structure [64]. Lima et al. [66] found that increased temperature changed the organization of cell wall polysaccharides in coffee leaves, and disordered cell wall microfibrils were observed in *Chara vulgaris* after exposure to cadmium and inorganic lead, which resulted in local wall protuberances [67]. Our results suggest that the interaction between stressors may be antagonistic for Cu accumulation, but further research is required to discriminate the mechanisms responsible for the reduced accumulation at increased temperatures.

## 4. Materials and Methods

### 4.1. Strain Selection

Previous research has shown that some strains of *Ectocarpus* collected from Cu-polluted sites are able to resist high Cu concentrations compared to strains collected at pristine sites [27,59,64,68]. Based on these studies, strain Es524 (Culture Collection of Algae and Protozoa accession number 1310/333), isolated from Caleta Palito (Chañaral, Chile), a coastal site known to be impacted by Cu pollution from mining activities, was chosen to assess the potential interactions with increased temperatures. The sea surface temperature in the Chañaral region varies between 15 and 22 °C in summer, with a mean of 18.3 °C (http://www.shoa.cl, accessed on 19 January 2018), and 13 and 15 °C in winter, with a mean of 14.1 °C.

### 4.2. Culture Conditions and Experimental Design

Stocks of Chilean *Ectocarpus* strain Es524 (CCAP 1310/333) stored long-term at 15 °C were used as inoculum for 10 L cultures in polycarbonate bottles containing sterile natural seawater (NSW) enriched with Provasoli medium [69]. Cultures were grown for 3 weeks in a controlled environment chamber (Sanyo MLR 350T, Osaka, Japan) at 15 °C, photosynthetic photon flux density of 45–50 μmol m^−2^ s^−1^ (Luxline plus cool white, Sylvania), 14:10 h light/dark cycle, and filtered aeration (Millex^®^ pore size 0.22 μm; Merck Millipore, Dublin, Ireland). Once sufficient biomass was obtained for experimentation, approximately 500 mg of fresh weight (FW) was transferred, in triplicate, into individual polycarbonate flasks containing 100 mL of sterile NSW. Additionally, 30 mg FW was placed in similar flasks to assess growth and photosynthesis. All flasks containing *Ectocarpus* were kept at 15 °C during 6 days for acclimation.

After the acclimation period, *Ectocarpus* biomass was exposed to one of eight treatments comprising a combination of temperature, 15 °C (control condition) or 25 °C (elevated temperature condition), and Cu concentration, 0 (control), 0.8 (low), 1.6 (medium), and 3.2 μM (high) of CuSO_4_·5H_2_O, for 8 days (Table 2).

Photosynthetic parameters, pigment, and metal content were measured after 6 days of exposure, and, to measure differences in growth, two extra days were allowed. The elevated temperature treatment was chosen as 4 °C above the maximum temperature recorded in summer at Chañaral (a northern region in Chile) from where strain Es524 was collected, according to the predicted ocean warming increases by the end of the century [10,13]. The Cu concentrations were chosen based on the results of previous trials showing that 3.9 μM Cu decreased the maximum quantum yield of photosystem II (*F_v_*/*F_m_*) after 6 days of exposure at 15 °C in strain Es524. Media were renewed every 2 days to avoid Cu depletion due to complexation by ligands released by algal cells [63].

### 4.3. Growth Rate

Relative growth rates (RGRs) were estimated volumetrically by using the ‘Wintrobe tube’ method: variations in volume (V) were recorded at the time of inoculation (t_0_) and after 8 days of exposure to treatments (t_1_) with the aid of hematocrit (Wintrobe, CE) tubes (Assistent^®^, Sondheim, Germany), as described by [39]. RGRs were calculated using the formula according to Yong et al. [70]:

RGR = [(Vt_1_/Vt_0_)^1/t^ − 1] ×100 (% d^−1^)

### 4.4. Photosynthesis and Energy Dissipation as In Vivo Chlorophyll a Fluorescence

In vivo chlorophyll *a* fluorescence of photosystem II (PSII) was measured with a MINI-PAM Photosynthesis Yield Analyzer (Walz, Effeltrich, Germany), according to Celis-Plá et al. [37] and Figueroa et al. [71]. To ensure the same dark-adaptation conditions and the same distance between probe and samples, pre-dawn measurements were performed by transferring each sample to a ‘dark leaf’ clip (DLC8, Walz) 5 min before measuring. Rapid light curves (RLCs) were created to obtain all photosynthetic parameters. The maximum photochemical quantum yield, *F_v_*/*F_m_*, was calculated as [72]

*F_v_*_/_*F_m_* = (*F_m_* − *F_o_*)/*F_m_*

where *F_o_* is the basal fluorescence, and *F_m_* is the maximal fluorescence of the dark-adapted samples obtained from the first saturating pulse (SP) of light (>4000 μmol m^−2^ s^−1^), which was the first step in the RLC. Following the first SP, the instrument automatically ran eight light incubations steps (20 s each) at increasing irradiances (E1 = 25, E2 = 76, E3 = 157, E4 = 249, E5 = 375, E6 = 508, E7 = 748, E8 = 1028 μmol m^−2^ s^−1^). An SP was applied at the end of the 20 s incubation at each irradiance level to measure basal (*F*) and maximal fluorescence (*F_m_ʹ*), which were used for the calculation of the effective quantum yield in the light-adapted state: Δ*F*/*F_m_ʹ*. The MINI-PAM was computer-operated by WinControl (Version 2.0, WALZ) software, supplied by the manufacturer.

The electron transport rate (ETR) at each irradiance level of RLC was calculated according to the formula

*ETR* (μmol electrons m^−2^ s^−1^) = Δ*F*/*F_m_ʹ* × *E_i_* × *A* × *F_II_*

where Δ*F*/*F_m_ʹ* is the effective quantum yield in the light-adapted state, *E_i_* is the incident irradiance (μmol m^−2^ s^−1^), *A* is the thallus absorptance, measured as the fraction of incident irradiance that is absorbed by the sample [54,71], and *F_II_* is the fraction of photons absorbed by the chlorophyll in PSII (400–700 nm), being 0.8 in brown seaweeds [71].

The curves resulting from plotting the *ETR* versus the incident irradiance were fitted to a model according to Eilers and Peeters [73] (Figure S1). The initial slope of the curve (*α*) and the maximum electron transport rate (*ETR_max_*) are estimators of photosynthetic efficiency, obtained from the tangential functions of the curve [74]. The minimum saturating irradiance (*E*_k_) was calculated from the intersection between *ETR_max_* and *α* [75].

The non-photochemical quenching NPQ at each irradiance level was calculated as

NPQ = (F_m_ − F_m_ʹ)/F_m_ʹ

Similarly, the curve resulting from the plot of NPQ versus irradiance was fitted to the same model [73] to calculate the maximum non-photochemical quenching (*NPQ_max_*) as an estimator of heat dissipation processes [76].

### 4.5. Pigments Content

Concentrations of chlorophyll *a* (Chl*a*) and *c* (Chl*c*) and fucoxanthin (Fx) were measured according to Seely et al. [77]. Fresh weight biomass (200 mg) was added to a centrifuge tube containing 800 μL of dimethyl sulfoxide (DMSO) (Sigma-Aldrich, Steinheim, Germany). After 5 min of incubation, samples were centrifuged at 21 000 *g_av_* (MSE-Sanyo, Heathfield, UK) for 1 min. The supernatant was diluted in a ratio of 4:1 of DMSO:water (800 μL + 200 μL _dd_H2O) and the absorbance measured at 665, 631, 582, and 480 nm with a spectrophotometer (Jenway 7315, Cole-Parmer, Stone, UK). Pigment concentrations (nmol g^−1^ DW) were calculated using the equations

$$\left[Chla\right]=\frac{A_{665}}{72.5}$$

$$\left[Chlc\right]=\frac{\left(A_{631}+A_{582}-{0.297A}_{665}\right)}{61.8}$$

$$\left[Fx\right]=\frac{\left(A_{480}-0.722\left(A_{631}+A_{582}-{0.297A}_{665}\right)-{0.049A}_{665}\right)}{130}$$

### 4.6. Metal Content Analysis

At the end of the experiment, 40 mg (FW) of biomass was taken from each replicate flask, excess water was removed, and the biomass was immediately frozen at −80 °C until analysis of Cu concentrations. To distinguish intracellular (non-exchangeable) concentrations from total Cu concentrations, another 40 mg FW of biomass was washed twice (15 min each time) in 2 mL NSW containing 10 mM EDTA to remove the exchangeable Cu fraction bound to cell walls [78]. Water excess was removed, and samples were stored at −80 °C. Frozen material was freeze-dried for 24 h and then acid-digested with 2 mL of 70% (*w*/*w*) HNO_3_ in a microwave oven (MARSXpress, CEM, Buckingham, UK). Digested samples were diluted to 5 mL with milli-Q water, and copper concentrations were determined by quadrupole inductively coupled plasma mass spectrometry (ICP-MS) X Series 2 (Thermo Scientific, Hemel Hempstead, UK). A calibration curve was prepared with pure Cu standards in 4% HNO_3_ (SCP science, Canada) at concentrations ranging from 0.05 ppm to 0.5 ppm diluted in milli-Q water. To validate the method, equal amounts of certified reference material (dried seaweed powder of *Fucus* spp., IAEA-140/TM) [79] were acid-digested and analyzed in the same way as the *Ectocarpus* experimental biomass. A Cu standard of known concentration was re-measured in intervals of 10 samples to control the calibration of the instrument. Cu concentrations were transformed to molarity and are reported on a dry weight basis.

### 4.7. Statistical Analysis

The effects of the treatments on the measured parameters were assessed using analysis of variance (two-way ANOVA) for the two fixed factors: (1) temperature (with two levels) and (2) copper concentration (with four levels), followed by Tukey’s post hoc tests. The Shapiro–Wilk and Levene’s tests were used on the datasets to assess the normality and homogeneity of variance, respectively. When assumptions of normality or homogeneous variance were not met, data were transformed using the square root function. The statistical package used was SPSS version 23 (IBM Corp., Armonk, NY, USA).

## 5. Conclusions

There is limited empirical information on the interactions between global environmental factors, such as ocean warming, and local pressures such as pollution by metals; however, there is a growing concern that interactions between these stressors will alter the responses of biota in unpredictable ways. Most findings so far suggest that elevated temperature does increase sensitivity to metals [26,80,81,82,83]. On the other hand, there is some evidence indicating that higher temperatures may increase the Cu tolerance in brown [49] and green seaweeds [53]. The results presented here provide valuable information to better understand the interactive effects of increased temperature and excess Cu in the stress response of *Ectocarpus*, suggesting that increased temperature helps to offset the negative impacts of exposure to high Cu concentrations. However, more in-depth studies assessing different algae species and metals as well as responses at other levels of biological organization would contribute to understanding the effects of global warming on metal metabolism in seaweeds.

## Figures and Tables

**Table 1:** Percentage (means) of intracellularly and extracellularly bound Cu ions in *Ectocarpus* after 6 days of exposure to combinations of Cu concentration (0, 0.8, 1.6 and 3.2 μM) and temperature: 15 °C and 25 °C. Standard deviations are shown in parenthesis.

Cu Treatment	Cu Accumulation (%)			
	15 °C		25 °C	
	Intracellular	Extracellular	Intracellular	Extracellular
Control	82.18 (6.9)	17.82 (6.9)	93.12 (14.6)	6.88 (14.6)
0.8 μM	73.47 (10.0)	26.53 (10.0)	83.28 (14.2)	16.72 (14.2)
1.6 μM	81.42 (9.9)	18.58 (9.9)	50.11 (6.2)	49.89 (6.2)
3.2 μM	63.36 (1.7)	36.64 (1.7)	58.12 (5.2)	41.88 (5.2)

**Table 2:** Experimental design and parameters measured.

Treatment	Temperature (°C)	Cu Concentration (μM)	Endpoints (*n* = 3)
T1	15	0	Day 6 of exposure: *F_v_*_/_*F_m_*; *α*; *ETR_max_*; *E_k_*; *NPQ_max_*, Pigments, Cu content Day 8 of exposure: RGR
T2	15	0.8	
T3	15	1.6	
T4	15	3.2	
T5	25	0	
T6	25	0.8	
T7	25	1.6	
T8	25	3.2	

## Data Availability

The original contributions presented in this study are included in this article/Supplementary Material. Further inquiries can be directed to the corresponding author.

## Supplementary Materials

The following supporting information can be downloaded at: https://www.mdpi.com/article/10.3390/plants14121834/s1, Table S1: ANOVA results showing the effects (significance level α = 0.05) of increased temperature and Cu excess. Figure S1. Rapid light curves resulting from plotting the ETR versus incident irradiance (PAR) and used to derive the parameters *α*, *ETR_max_*, *E*_k_ at (a) 15 °C and (b) 25 °C; rapid light curves resulting from plotting NPQ versus incident irradiance (PAR), used to derive the parameter *NPQ_max_* at (c) 15 °C and (d) 25 °C.

## Abbreviations

The following abbreviations are used in this manuscript:

*α*	Efficiency of light captured for photosynthesis
CCAP	Culture Collection of Algae and Protozoa
Chl*a*	Chlorophyll *a*
Chl*c*	Chlorophyll *c*
*E*_k_	Minimum saturating irradiance
*ETR_max_*	Maximum electron transport rate
*F*	Basal fluorescence in the light-adapted state
*F_o_*	Basal fluorescence in the dark-adapted state
*F_m_*	Maximal fluorescence of the dark-adapted
*F_m_’*	Maximal fluorescence of the light -adapted
Δ*F*/*F_m_ʹ*	Effective quantum yield in the light-adapted state
*F_v_*/*F_m_*	Maximum quantum yield
FW	Fresh weight
Fx	Fucoxanthin
MHWs	Marine heat waves
NPQ	Non-photochemical quenching
*NPQ_max_*	Maximal non-photochemical quenching
NSW	Natural seawater
PAR	Photosynthetically active radiation
PSII	Photosystem II
RGR	Relative growth rate
RLC	Rapid light curve
ROS	Reactive oxygen species
RuBisCO	Ribulose-l,5-bisphosphate carboxylase/oxygenase
SST	Sea surface temperature
SP	Saturating pulse of light
