# Supplementary material for: Copper and Temperature Interactions Induce Differential Physiological and Metal Exclusion Responses in the Model Brown Macroalga Ectocarpus
Source: Plants (Basel). 2025 Jun 14;14(12):1834. doi: 10.3390/plants14121834 (PMC12196947; doi:10.3390/plants14121834)

Figure S1. Rapid Light Curves (RLCs) resulting from plotting the ETR versus incident irradiance (PAR) and used to derive the parameters: α, ETR_{max}, E_k at a) 15°C and b) 25°C; and RLCs resulting from plotting the NPQ versus PAR, used to derive the parameter NPQ_{max} at c) 15°C and d) 25°C.

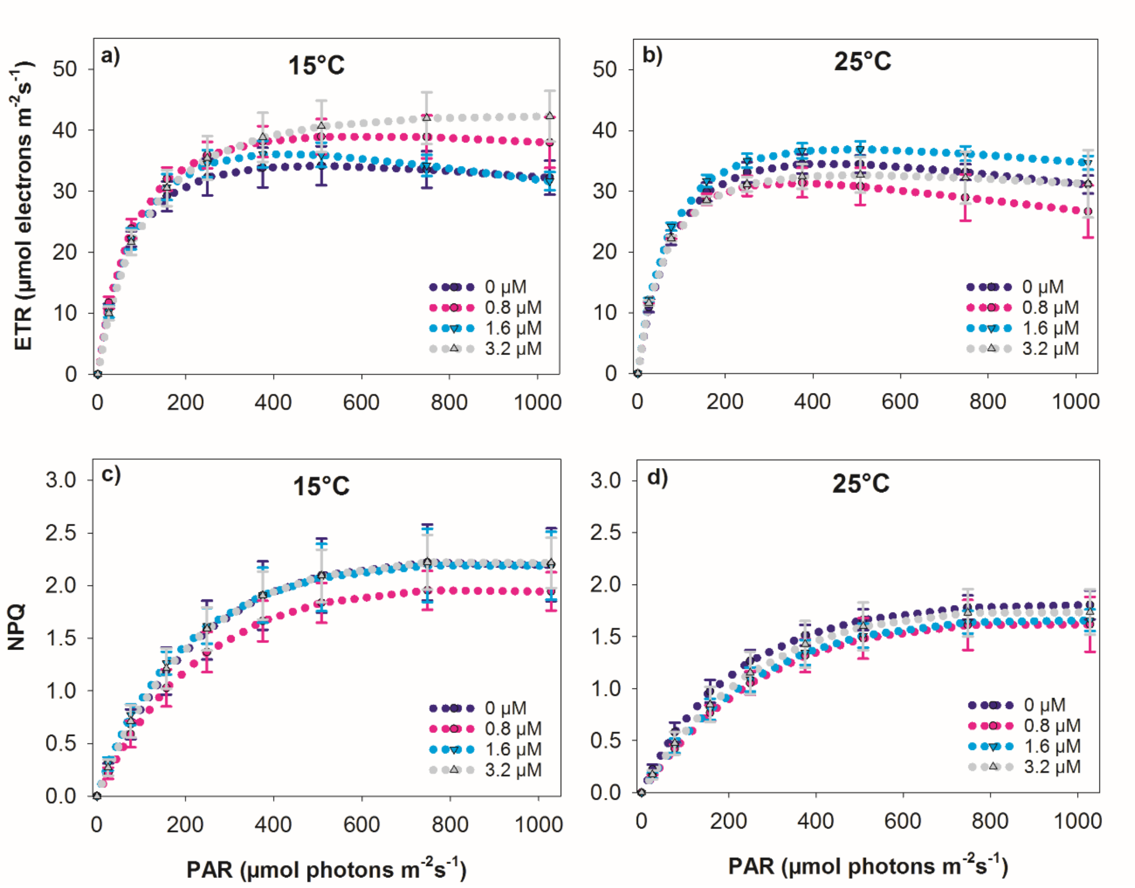

Supplement: Supplementary file 1 [file plants-14-01834-s001.zip › Figure S1.pdf]
