# Supplementary material for: Copper and Temperature Interactions Induce Differential Physiological and Metal Exclusion Responses in the Model Brown Macroalga Ectocarpus
Source: Plants (Basel). 2025 Jun 14;14(12):1834. doi: 10.3390/plants14121834 (PMC12196947; doi:10.3390/plants14121834)
Supplement: Supplementary file 1 [file plants-14-01834-s001.zip › Table S1.pdf]

Table S1. ANOVA results showing the effects (significance level α = 0.05) of increased temperature and Cu excess on: **a)** relative growth rate; **b)** photosynthetic parameters; **c)** pigments content; and **d)** copper accumulation in *Ectocarpus* sp. strain Es524.

	Source of variation	df	MS	*F*	*P*		Source of variation	df	MS	*F*	*P*
**a)**											
**RGR**	Cu	3	0.68	1.74	0.199						
	Temperature	1	9.05	**23.13**	**<0.001**						
	Cu × Temperature	3	0.59	1.50	0.252						
	Residual	16	0.39	0.39							
**b)**											
F_v/F_m	Cu	3	0.00048	2.43	0.103	α	Cu	3	0.0027	1.08	0.384
	Temperature	1	0.00197	**10.06**	**0.006**		Temperature	1	0.0273	**10.82**	**0.005**
	Cu × Temperature	3	0.00034	1.731	0.201		Cu × Temperature	3	0.0133	**5.26**	**0.010**
	Residual	16	0.0002				Residual	16	0.0025		
ETR_{max}	Cu	3	18.51	2.50	0.097	E_k	Cu	3	203.67	2.85	0.070
	Temperature	1	112.0	**15.11**	**0.001**		Temperature	1	1466.4	**20.53**	**<0.001**
	Cu × Temperature	3	49.78	**6.72**	**0.004**		Cu × Temperature	3	454.81	**6.37**	**0.005**
	Residual	16	7.41				Residual	16	71.44		
NPQ_{max}	Cu	3	0.059	0.97	0.430						
	Temperature	1	1.191	**19.66**	**<0.001**						
	Cu × Temperature	3	0.013	0.212	0.886						
	Residual	16	0.061								
**c)**											
**Chl *a***	Cu	3	50.43	0.75	0.537	**Chl *c***	Cu	3	406.8	0.63	0.607
	Temperature	1	35.19	0.52	0.479		Temperature	1	102.9	0.16	0.695
	Cu × Temperature	3	152.63	2.28	0.119		Cu × Temperature	3	1185.4	1.83	0.182
	Residual	16	66.99				Residual	16	647.3		
**Fx**	Cu	7	5150.77	**3.43**	**0.042**						
	Temperature	1	26730.7	**17.80**	**<0.001**						
	Cu × Temperature	7	4045.47	2.69	0.081						
	Residual	32	1501.59								
**d)**											
**Intracellular**	Cu	3	4.136	**113.7**	**<0.001**	**Total**	Cu	3	14.38	**206.8**	**<0.001**
	Temperature	1	0.663	**18.23**	**<0.001**		Temperature	1	0.384	**5.52**	**0.032**
	Cu × Temperature	3	0.342	**9.41**	**<0.001**		Cu × Temperature	3	0.426	**6.12**	**0.006**
	Residual	16	0.036				Residual	16	0.07		

F_v/F_m: maximum quantum yield; α: efficiency of light capture for photosynthetic; ETR_{max}: maximum electron transport rate; E_k: minimum saturating irradiance; NPQ_{max}: maximal non-photochemical quenching; **Chl*a***: Chlorophyll *a*; **Chl*c***: Chlorophyll *c*; **Fx**: Fucoxanthin; **RGR**: relative growth rate.
